# Risk Factors for Outbreaks of Lumpy Skin Disease and the Economic Impact in Cattle Farms of Nakuru County, Kenya

**DOI:** 10.3389/fvets.2020.00259

**Published:** 2020-05-29

**Authors:** Samuel Kipruto Kiplagat, Philip Mwanzia Kitala, Joshua Orungo Onono, Philippa M. Beard, Nicholas A. Lyons

**Affiliations:** ^1^Department of Public Health, Pharmacology and Toxicology, University of Nairobi, Nairobi, Kenya; ^2^The Pirbright Institute, Pirbright, United Kingdom; ^3^The Roslin Institute, University of Edinburgh, Midlothian, United Kingdom; ^4^European Commission for the Control of Foot-and-mouth Disease, Food and Agriculture Organization of the United Nations, Rome, Italy

**Keywords:** economic impact, risk factors, lumpy skin disease, case-control study, vaccine

## Abstract

Lumpy Skin Disease (LSD) is an emerging disease of cattle that causes substantial economic loss to affected regions. However, factors favouring transmission under field conditions and farm-level impacts are poorly quantified. This was a retrospective case-control study of cattle farms in Nakuru, Kenya to determine risk factors associated with lumpy skin disease and the farm-level economic impacts of an outbreak. Data were collected using questionnaires administered through personal interview. Collected data included herd sizes, age, and sex structures, breeds, sources of replacement stock, grazing systems, and costs (direct and indirect) incurred when LSD outbreaks occurred. Farm-level risk factors were examined through univariable and multivariable logistic regression and a final model built using backward stepwise regression and likelihood ratio tests. The factors associated with LSD outbreaks on univariable analysis included breed (exotic vs. indigenous, OR = 15.01, *P* = 0.007), source of replacement stock (outside the herd vs. within the herd, OR = 8.38, *P* < 0.001) and herd size (large [>10 cattle] vs. small [1–3 cattle], OR = 3.51, *P* = 0.029). In the multivariable logistic regression model, only breed (exotic vs. indigenous, OR = 14.87, 95% CI 1.94–113.97, *P* = 0.009) and source of replacement stock (outside the herd vs. within the herd OR = 8.7, 95% CI 2.80–27.0, *P* < 0.001) were associated with outbreaks. The economic impact was compared between farms keeping purely indigenous (*n* = 10) or exotic (*n* = 29) breeds of cattle which indicated mean farm-level losses of 12,431 KSH/123 USD and 76,297 KSH/755 USD, respectively. The mean farm-level losses from reduction in milk yield and mortality were estimated at 4,725 KSH/97 USD and 3,103 KSH/31USD for farms keeping indigenous breeds whilst for farms keeping exotic breeds the equivalent losses were 26,886 KSH/266 USD and 43,557 KSH/431 USD, respectively. The indirect losses from treatments and vaccinations were proportionately much higher on farms with indigenous breeds at 4,603 KSH/46 USD making up ~37% of the total costs compared to ~8% (5,855 KSH/58 USD per farm) of the total costs for farms with exotic breeds. These findings indicate that LSD caused significant economic losses at the farm level in Nakuru County. This justifies implementation of disease control measures including quarantine of cattle post-purchase and the need for effective vaccinations of susceptible cattle herds.

## Introduction

Lumpy skin disease (LSD) is caused by the poxvirus lumpy skin disease virus in genus *Capripoxvirus* that also includes the closely related sheeppox virus and goatpox virus (1–3). Cattle are the predominant species affected although infection has also been reported in water buffalo (4, 5). Historically, the virus was restricted to the African continent with sporadic incursions into the Middle East, but since 2012 the disease has spread in easterly and westerly directions as far as the Balkans and Kazakhstan (6, 7).

There have been several other studies of LSD in the region particularly in Ethiopia. In West Wolega, Zelalem et al. (8) estimated individual and herd-level seroprevalence of 6.4 and 6.0%, respectively. Their study demonstrated relatively higher seroprevalence in older animals and in *Bos taurus* compared to *Bos indicus* cattle. Also in Ethiopia, it was reported that disease burden was lower in highland compared to mid- and lowland areas possibly related to the relative abundance of biting fly populations (9–11). Communal grazing and watering has also been associated with a higher occurrence of LSD, likely due to the increased opportunity for mechanical transmission of the virus by *Stomoxys* spp. and mosquitoes (*Aedes aegypti*) (9, 12, 13). In Zimbabwe, higher LSD was associated with the proximity to game parks suggesting the wildlife-cattle interface may be important for transmission (14). Numerous wildlife hosts have been suspected including the African Cape Buffalo (15, 16). Transhumance and other reasons for animal movements has also been associated with an increased risk of outbreaks (17, 18).

Clinical signs of LSD include the appearance of raised, circular, firm, coalescing nodules on the skin which can develop cores of necrotic material called “sit-fasts” (19). Lumpy Skin Disease virus (LSDV) is thought to be primarily transmitted by biting and blood feeding arthropods that include species from the *Glossina, Muscidae*, and *Tabanidae* families, in addition to some species of hard tick (11, 20–23, 23–26). Virus transmission through direct contact has been reported but this is considered an inefficient route (27–29). LSD is a listed disease according to the World Animal Health Organization (OIE) due to the potential for rapid spread of virus in susceptible cattle populations and the severe economic consequences it causes in affected herds (30).

In the event of an incursion into a previously LSDV-free country, control measures may include strict quarantine, restriction of animal movements, reactive vaccination, isolation and slaughter of affected animals, proper disposal of carcasses, cleaning and disinfection of the premises and insect control (11, 29, 31). Occasionally, whole herd depopulation has been recommended, but in endemic scenarios the affected farms often isolate sick animals and provide supportive treatment that may include wound dressings to prevent fly infestations and secondary infections (11, 29). Vaccination may also be used with both reactive and routine strategies being employed. Most currently available vaccines are live attenuated and contain either LSDV (homologous) or sheep and goat pox strains (heterologous) (11, 31). Heterologous vaccines are generally considered to be less effective but with fewer side effects particularly in European breeds of cattle (32).

It has been proposed that the control of LSD is likely to enhance the livelihoods of farmers and others dependent on livestock (33). Although LSD outbreaks are generally associated with lower morbidity and mortality rates in herds when compared to some other OIE listed livestock diseases, the economic consequences of the outbreak results from prolonged loss of production in both dairy and beef cattle through loss of weight in diseased cattle, and loss of traction for farms using cattle as a source of draught power (11, 34). Other types of direct losses may include reduced quality of hides and meat, reduced milk yield in affected herds, culling of affected animals, and infertility secondary to severe orchitis (2, 11, 35). Indirect losses are either from additional costs of disease control or the value of foregone revenue. Additional costs may be from vaccines, vaccine delivery, movement controls, use of diagnostic tests, and culling animals while the value of foregone revenue include use of sub-optimal breeds and denied access to both local and international markets (36). Studies estimating farm-level losses due to LSD outbreaks in endemic settings are lacking in the literature although a recent study in Ethiopia estimated the median total economic loss of an LSD outbreak at herd level to be USD 1,176 with the largest component due to mortality followed by reduction in milk output (37).

Lumpy Skin Disease is endemic throughout East Africa and was first described in Kenya in 1957 (38). Since then, LSD epidemics have been reported irregularly in various parts of Kenya (39). Despite the assumed importance of disease, no studies have been published describing the risk factors associated with its occurrence at the farm level and the economic costs incurred by livestock keepers in Kenya. These studies are essential for informing control strategies and allocating limited resources for livestock disease control at a farm and national level. The aim of this study was to determine the risk factors for LSD outbreak through a matched case-control study and to estimate the economic impact on affected farms.

## Materials and Methods

### Study Area and Population

**Figure 1:**
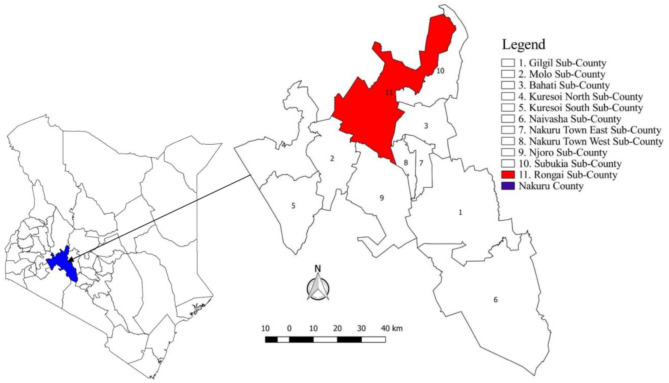
Map of Kenya showing the location of Nakuru County and 11 Sub-counties.

The study was undertaken in Nakuru County, Kenya, an area of 7,495 km^2^ with 11 administrative Sub-counties (Figure 1). According to the previous national census, the County was the fourth most populous in the country with ~1.6 million people. The same census revealed a livestock population of 439,994 cattle, 505,035 sheep, and 227,037 goats (40). There are a variety of production systems present in Nakuru County including pastoralism, intensive, and semi-intensive. Pastoralists were not included in this study due to their extensive movements making retrospective assessments of risk factors and choice of controls extremely challenging. The eligible population for inclusion in this study was all sedentary cattle herds present in the County between September 2016 and October 2017. The county was selected due to a pre-existing network of collaborators, frequent reports of disease and the importance of cattle in the farming systems.

### Study Design and Sample Size Determination

The study utilised an individual-matched case-control approach due to the reported low incidence of disease based on local expert opinion. The sampling units were households that kept cattle. Potential case farms were initially identified through discussions with Sub-county Veterinary Officers (SCVOs) and their staff including a review of written records of attended cases and rumoured outbreaks. Subsequent case herds were identified through discussion with local animal health practitioners and farmers affected with disease. Unaffected households (controls) were selected by assigning all non-case households in the same village with a unique identification number and randomly selecting until the desired number of control households was reached. The sample size was estimated using the epiR (version 0.9-99) package in R3.5.2 based on the methodology described by Dupont (41) in 1988 (Equation 1). This used the following equation to estimate the sample size for frequency matched case control studies:

(1) $$N=\frac{{\left(z_{\beta }v_{\varphi }^{\frac{1}{2}}+z_{\frac{\alpha }{2}}v_{1}^{\frac{1}{2}}\right)}^{2}}{{\left(e_{1}-e_{\varphi }\right)}^{2}}$$

ψ = Odds ratio for exposure in case and controls.

v = Variance

e = Exponent

Z = Number of standard deviations from the data point mean

α = Type I error probability

β = Type II error probability

*N* = Number of cases

To optimise the efficiency of the study, four controls were matched for each case. Based on the assumption of 20% controls having a risk factor of interest, in order to have 80% power to detect an odds ratio (OR) of 3.0 with 95% confidence, 41 cases and 164 controls were required. This calculation assumed a moderate correlation in exposures between case and control exposures (rho = 0.2).

### Case and Control Definitions

Case herds were defined based on clinical suspicion of LSD in at least one bovine demonstrating the characteristic clinical sign of raised, circular, firm, nodules varying from 1 to 7 cm in diameter (11, 29, 42). Case farms were eligible for recruitment if the suspected case occurred between September 2016 and October 2017. Laboratory confirmation was not readily available, so case farms were based on clinical suspicions only. Matching criteria for control herds were based within the same village and not reporting suspected LSD between September 2016 and October 2017.

### Data Collection

Primary data were collected on household-level herd structures and putative risk factors for LSD between October 2017 and February 2018. Household-level risk factors were chosen based on those described in the literature and included breed, introduction of new animals, vaccination status against LSD (pre-LSD outbreak and post-LSD outbreak vaccination was recorded and confirmed with the sub-county veterinary department), and management practices that encouraged inter-herd contact (including using communal grazing and watering points, mixing in post-harvest fields, communal dipping for acaricide administration, and breeding system). A questionnaire was created and administered using KoboCollect® mobile phone application. The questionnaire was piloted with five households in September 2017 to detect any questions that could be ambiguous. Apart from questions on direct and indirect losses from case households, the questionnaire was identical for case and control herds and is available as Supplementary Material to this manuscript.

The economic impact was estimated based on the framework described by Rushton et al. (36), where direct losses are incurred through reduction on level of production and indirect losses incurred through reactions to disease occurrence either by treatment or application of prevention measures. Direct losses were estimated from the increased number of cattle mortalities and reduced milk production in affected cattle herds, while indirect losses were estimated from the costs incurred on preventive vaccination and treatment of sick cattle. The cost of cattle mortality considered the number, age, and sex of those affected herds, and prices of livestock and their products were obtained from various livestock markets and farms breeding cattle for sale. Furthermore, this information from the farmers was verified with local animal health providers who attended to the cases. Losses in milk were estimated from the reduction in level of milk production and duration a herd remained with clinical disease as reported by the interviewed farmers. The cost of vaccination was based on the fees (Ksh) households were charged for vaccinating their cattle against LSD (which may or may not have been subsidised by the County government). While treatment costs were based on the fees (Ksh) charged on households for treatment of clinical cases of LSD including the purchase price for antibiotics and other transactional costs including consultation and transport charges by the animal health services provider.

### Data Management and Analysis

Data were downloaded from KoboCollect® mobile application and exported to MS Excel® 2010 prior to analysis using Stata 13® (StataCorp, College Station, TX, USA). The data analysis involved estimating descriptive statistical measures and inferential statistics.

Logistic regression was used to generate odds ratios and estimate the strength of evidence at the household-level between putative risk factors and having at least one case of LSD. All models included the matching variable (village) as a fixed effect. Variables associated on univariable logistic regression analysis (at *P* ≤ 0.2) were taken forward for possible inclusion in a multivariable model. This conservative *P*-value was used in the univariable analysis to include as many variables as possible in the multivariable analysis. The final multivariable model was built using a backward stepwise approach and likelihood ratio tests (LRT) to compare models with and without each of the variables. Variables were retained if the *P*-value of the LRT was ≤ 0.05. Interaction between variables in the final model was tested using LRTs and the presence of collinearity was tested by estimating variance inflation factors. The economic losses were compared between farms keeping indigenous and exotic breeds using unpaired *t*-tests. The approach used by Jemberu et al. (43) in estimation of economic impact of Foot and Mouth Disease and by Molla et al. (37) in estimation of economic impact of LSD in Ethiopia was adopted in the analysis of the estimated economic losses.

The economic cost of LSD vaccination was calculated as;

(2) $$\begin{array}{r} \text{Vacos}{\text{t}}_{\text{ij}}\;=\text{ NV}{\text{a}}_{\text{i}}^{*}\text{PV}{\text{a}}_{\text{i}} \end{array}$$

Where,

Vacost_ij_ = the vaccination cost for affected herd i with breed j (without consideration of subsidy if any);

NVa_i_ = the number of animals vaccinated;

PVa_i_ = the mean per head expenditure on LSD vaccination (whether prior or post LSD);

The economic cost of LSD treatment was calculated as;

(3) $$\begin{array}{r} \text{TrCos}{\text{t}}_{\text{ij}}\;=\text{ NT}{\text{r}}_{\text{i}}^{*}\text{PT}{\text{r}}_{\text{i}} \end{array}$$

Where,

TrCost_ij_ = the treatment cost for affected herd i with breed j;

NTr_i_ = the number of animals treated;

PTr_i_ = the mean per head expenditure to LSD treatment;

Economic losses due to milk loss per LSD affected herd were calculated as;

(4) $$\begin{array}{r} \text{Lmil}{\text{k}}_{\text{ij}}\;=\text{ Nco}{\text{w}}_{\text{i}}^{*}{\text{Q}}_{\text{i}}^{*}\text{Tmil}{\text{k}}_{\text{i}}^{*}\text{Pmil}{\text{k}}_{\text{j}} \end{array}$$

Where,

Lmilk_ij_ = economic losses due to milk loss for herd i with breed j;

Ncow_i_ = number of lactating cows affected in herd i;

Q_i_ = mean quantity of milk lost in liters per affected herd per day in herd i;

Tmilk_i_ = mean duration of illness in days of affected lactating cows in herd i,

Pmilk_j_ = mean selling price of milk per litre reported by farmers in herd i. The economic loss due to mortality per herd was calculated as

(5) $$\begin{array}{l} {\text{Lmort}}_{\text{ij}}=\left({\text{Nmortfcalf}}_{\text{i}}^{*}\text{Pfcalf}\right)\\ \;+\left({\text{Nmortmcalf}}_{\text{i}}^{*}\text{Pmcalf}\right)\text{+}\left({\text{Nmortheif}}_{\text{i}}^{*}\text{Pheif}\right)\\ \;+\left({\text{Nmortbull}}_{\text{i}}^{*}\text{Pbull}\right)\text{+}\left({\text{Nmortlact}}_{\text{i}}^{*}\text{Plact}\right)\\ \;+\left({\text{Nmortdry}}_{\text{i}}^{*}\text{Pdry}\right) \end{array}$$

Where,

Lmort_ij_ = economic losses due to mortality for a herd i with breed j;

Nmortfcalf_i_ = number of female calves died in herd i;

Pfcalf = price of a female calf;

Nmortmcalf_i_ = number of male calves died in herd i;

Pmcalf = price of a male calf;

Nmortheif_i_ = number of heifers died in herd i;

Pheif = price of a heifer;

Nmortbull_i_ = number of bulls died in herd i;

Pbull = price of a bull;

Nmortlact_i_ = number of lactating cows died in herd i;

Plact = price of a lactating cow;

Nmortdry_i_ = number of dry cows died in herd i;

Pdry = price of a dry cow;

Total economic losses per herd were aggregated as the sum of all losses arising from milk loss, mortality, cost of treatment and cost of vaccination.

(6) $$\begin{array}{r} \text{TE}{\text{L}}_{\text{ij}}\;=\text{ Vacos}{\text{t}}_{\text{ij}}^{*}\text{TrCos}{\text{t}}_{\text{ij}}^{*}\text{Mil}{\text{k}}_{\text{ij}}^{*}\text{Mor}{\text{t}}_{\text{ij}} \end{array}$$

Where,

TELij = total economic losses for herd i in a farm with breed j,

The mean economic loss per head of cattle was obtained by dividing the herd-level economic losses by the total number of cattle in the herd. The mean of each of the economic losses per affected herd was obtained by dividing the specific economic losses in the herd by the total number of herds affected.

All KSH to USD conversion rates are the yearly means as per the central bank of Kenya forex rates (available at https://www.centralbank.go.ke/rates/forex-exchange-rates/) for the year in which a study was conducted.

Ethical approval for the study was obtained through the Biosafety, Animal Care and Use committee within the Faculty of Veterinary Medicine at the University of Nairobi, Kenya (Reference number FVM BAUEC/2018/171).

## Results

### Characteristics of the Study Farms

Of the 11 sub-counties in Nakuru County, one (Rongai) reported cases during the study period with the others reporting cases prior to the study inclusion dates. A total of 41 case farms and 164 control farms were visited in six villages of Rongai sub-county. The majority (165/205, 80.5%) of respondents were the farm owners. Other respondents were farm managers (17.1%) or other family members (2.4%) of the farm owner such as the wife and children. The mean herd sizes in both case and control farms was eight (range 1–69). In the case farms, the mean herd size was 11 (range 2–59) while in the control farms was 7 (range 1–69). This comprised of a mean herd size of 10.6 (range 1–59) for indigenous breeds of cattle and 5.6 (range 1–36) for exotic breeds of cattle.

The distribution of potential risk factors for LSD outbreaks in case and control herds is presented in Table 1. Based on univariable analysis, there was good statistical evidence that compared to non-affected control herds, case herds tended to consist of exotic (i.e., European) breeds (OR = 15.01, 95% CI 2.09–108.04), be larger in size (OR = 3.51, 95% CI 1.14–10.83), and source replacement cattle from outside the farm (OR = 8.38, 95% CI 2.93–23.92) as shown in Table 1. There was weak statistical evidence that case farms tended to use rivers as a communal water source (OR = 3.40, 95% CI 0.83–13.84), and use a communal dip as opposed to spraying at home for tick control (OR = 3.71, 95% CI 0.80–17.29). There was no statistical evidence of any difference in grazing system (OR = 2.88, 95% CI 0.31–26.74) and vaccination status (OR = 1.52, 95% CI 0.49–4.71) in case and control herds.

**Table 1:** Univariable analysis of household-level putative risk factors for Lumpy Skin Disease in Nakuru County, Kenya.

**Variable**	**Category**	**Cases (*n*)**	**%^**c**^**	**Control (*n*)**	**%^**d**^**	**OR**	**95% CI**		***P*-value**
Breed	Exotic	29	71	102	62	15.01	2.09	108.04	0.01
	Indigenous	10	24	59	36	Reference	–	–	–
	Mixed	2	5	3	2	15.50	0.03	1.39	172.83
Cattle herd size (categorical)	Small (1–3)	11	27	61	37	Reference	–	–	–
	Medium (4–9)	15	37	67	41	1.76	0.67	4.64	0.25
	Large (≥10)	15	37	36	22	3.51	1.14	10.83	0.03
Cattle herd size (continuous)		41	20	164	80	1.05	1.01	1.09	0.02
Dipping system	Home spraying	37	90	155	97	Reference	–	–	–
	Community dip	4	10	5	3	3.71	0.80	17.29	0.01
Breeding system	AI or own bull^a^	24	59	97	60	Reference	–	–	–
	Shared bull	17	42	66	41	1.11	0.40	2.36	0.84
LSD vaccination^b^	Yes	5	12	15	9	1.52	0.49	4.71	0.47
	No	36	88	149	91	Reference	–	–	–
Replacement cattle	From own herd	30	73	157	96	Reference			
	From outside	11	27	7	4	8.38	2.93	23.92	<0.01
Watering system	In rivers	11	27	29	18	3.40	0.83	13.84	0.09
	Communal dams	4	10	38	23	1.25	0.34	4.55	0.74
	Communal boreholes	0	0	1	1	1.00	–	–	–
	Piped and harvested water	26	63	96	59	Reference	–	–	–
Grazing system	Tethering	1	2	9	56	Reference	–	–	–
	Zero-grazing	30	73	118	72	2.60	0.31	21.94	0.38
	Free-range	10	24	37	23	2.88	0.31	26.74	0.35

*Odds ratios estimated using logistic regression. Reference categories are based on the largest category size except for “herd size” where the reference is the smallest farm size (1–3 cattle). AI, Artificial insemination; OR, odds ratio; CI, confidence interval*.

a *AI and own bull was combined as only 2.4% (4/164) of the control farms used own bull*.

b *LSD vaccination between January 2016 and October 2017*.

c *Calculation of the cases percentage = number of observations in that variable level divided by the total number of cases, which was 41*.

d *Calculation of the controls percentage = number of observations in that variable level divided by the total number of controls, which was 164*.

The multivariable analysis included all variables in the univariable models that had a *P*-value < 0.2. Backward fitting of the model using likelihood ratio tests and an inclusion cut-off of <0.05 led to two variables being retained in the final model. Farms with LSD were significantly more likely to source replacement cattle from outside the herd (outside sourced vs. own farm sourced OR = 8.7; 2.8–27.0, *P* < 0.01) and to consist entirely of exotic breeds (indigenous breed vs. exotic breed herds OR = 0.06; 0.01–0.52, *P* = 0.01) (Table 2). The result of variance inflation factors analysis showed moderate collinearity between breed and village which was included in all models as a fixed effect. However, dropping the breed variable from the model did not significantly affect the association between LSD status and the source of replacement cattle.

**Table 2:** Multivariable analysis of the risk factors of Lumpy Skin Disease Outbreaks in Nakuru County.

**Variable**	**Category**	**Case (*n*)**	**%**	**Control (*n*)**	**%**	**OR**	**95% CI**		***P*-value**
Replacement cattle	From own herd	30	73	157	96	Reference	–		–
	From outside	11	27	7	43	8.70	2.80	26.98	<0.01
Breed	Exotic breeds	29	71	102	62	14.87	1.94	113.97	0.01
	Indigenous	10	24	59	36	Reference	–		–
	Mixed breeds	2	5	3	2	7.05	0.52	95.98	0.14

*Village was the matching variable and included in the model as a fixed effect*.

On the farmers' knowledge of the disease, most of the farmers (*n* = 108, 78.8%) did not have any understanding of the reasons for LSD occurring on their farm. The remaining farmers believed the reasons were mixing of affected cattle with unaffected ones during roadside grazing (*n* = 6, 4.4%), spread by the wind (*n* = 5, 3.7%), spread by biting flies (*n* = 5, 3.7%), spread from the initial case that occurred in the area (*n* = 3, 2.2%), pastoralist cattle from Narok county in search of pasture (*n* = 2, 1.5%), and cattle passing through the area from Loruk in Baringo and Pokot Counties being taken for sale in Marigat, Mogotio, Nakuru, and Kenya Meat Commission within Nakuru county (*n* = 1, 0.7%) .

### Estimated Economic Impact of Lumpy Skin Disease in a Herd

**Figure 2:**
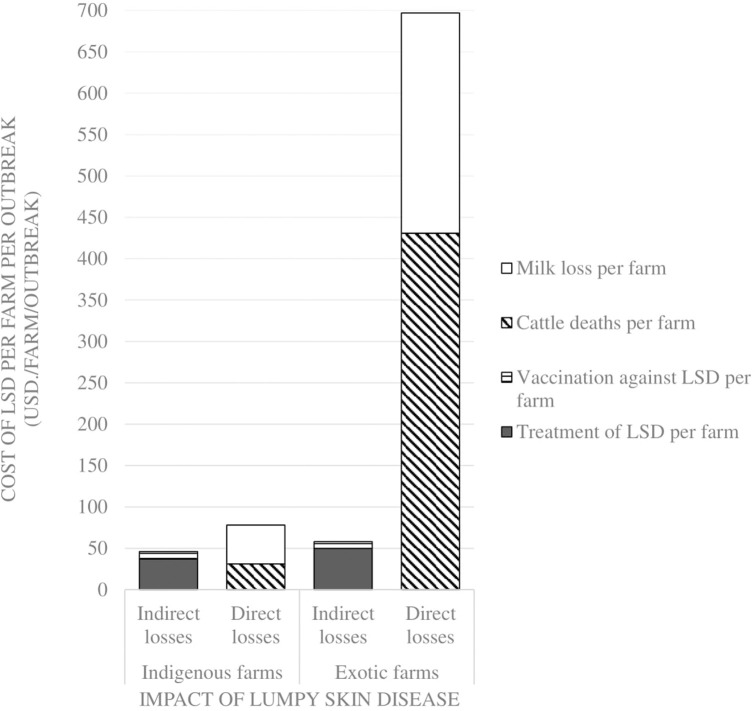
Comparison of the economic impact of Lumpy Skin Disease outbreaks between farms with only indigenous breeds vs. farms with only exotic breeds in Nakuru County, Kenya.

**Figure 3:**
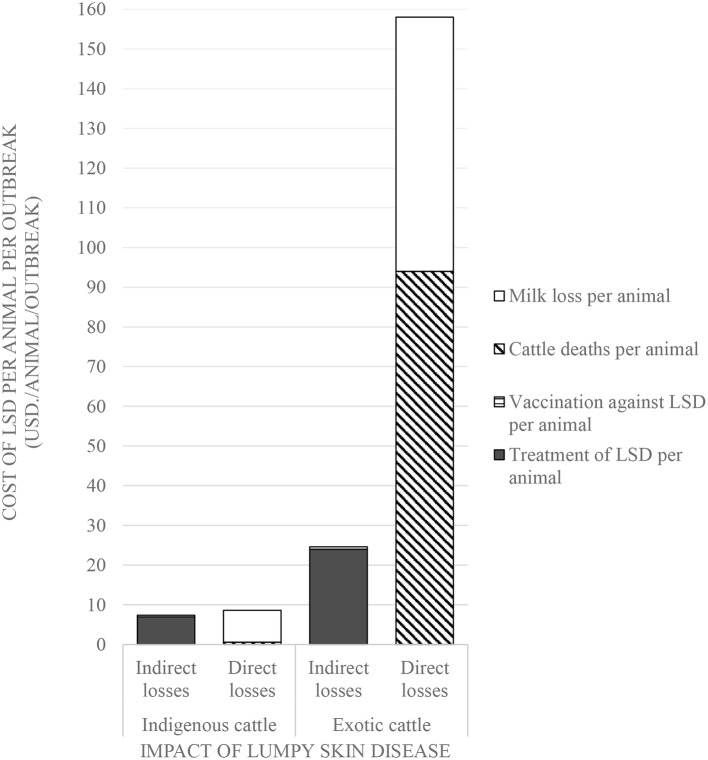
Comparison of the economic impact of Lumpy Skin Disease outbreaks between indigenous and exotic breeds of cattle in Nakuru County, Kenya.

Comparisons on the economic cost of LSD was compared in herds consisting of purely indigenous (*n* = 10) or exotic (*n* = 29) breeds of cattle. Two herds had mixed breeds and were excluded from the economic analysis). Considering direct costs, the reduction in milk production during LSD outbreaks was estimated at a mean of 1.5 l (Range 0–4) per farm per day for farms keeping indigenous cattle and 9.9 l (Range 0–35 l) per farm per day for farms keeping exotic breeds of cattle (*P* = 0.06). Based on an estimated duration of milk reduction of 70 days as reported in Ethiopia (37), this was equivalent to a mean total loss of 4,725 KSH/47 USD ranging from 2,520 to 10,080 KSH/25–100 USD milk reduction loss per farm for those keeping indigenous breeds of cattle. In comparison, the estimated mean total loss due to reduced milk production on farms keeping exotic breeds of cattle was 26,886 KSH/266 USD ranging from 2,520 to 88,200 KSH/25–873 USD (*P* = 0.15) as shown in Figure 2 and Table 3. The per head of cattle loss was equivalent to a mean loss of 831 KSH/8 USD (range 0–3,780 KSH/37 USD) and 6,440 KSH/64 USD (range 0–88,200 KSH/0–873 USD) for indigenous and exotic breeds of cattle respectively (*P* = 0.33) as shown in Figure 3 and Table 4.

**Table 3:** Losses incurred by farms due to LSD outbreak in Nakuru County (1USD=101KSH; two farms having mixed breeds were excluded in this analysis) per farm.

**Losses**	**Item of loss**	**Number of farms affected**	**Mean cost (KSH)**	**Standard deviation**	**Minimum cost (KSH)**	**Maximum cost (KSH)**
Direct losses	Mortality losses per affected indigenous cattle farm	2	3,103	4,134	180	6,026
	Mortality losses per affected exotic cattle farm	7	43,557	21,651	3,000	63,900
	Milk losses per affected indigenous cattle farm	4	4,725	3,619	2,520	10,080
	Milk losses per affected exotic cattle farm	13	26,886	28,876	2,520	88,200
Indirect losses	Cost of treatment of LSD in indigenous case farms	8	3,715	5,572	250	17,100
	Cost of treatment of LSD in exotic case farms	29	5,003	6,120	400	32,000
	Cost of vaccination of LSD in indigenous case farms	7	888	1,160	270	3,500
	Cost of vaccination of LSD farms with exotic breeds	13	852	1,732	90	6,480

**Table 4:** Losses incurred by farms due to LSD outbreak in Nakuru County (1 USD = 101 KSH; two farms having mixed breeds were excluded in this analysis) per head of cattle.

**Losses**	**Item of loss**	**Number of animals affected**	**Mean cost (KSH)**	**Standard Deviation**	**Minimum cost (KSH)**	**Maximum cost (KSH)**
Direct losses	Mortality losses per affected indigenous cattle	2	55	66	8	102
	Mortality losses per affected exotic cattle	7	9,485	6,953	500	19,133
	Milk losses per affected indigenous cow	9	831	1,318	0	3,780
	Milk losses per affected exotic cow	29	6,440	16,693	0	88,200
Indirect losses	Cost of treatment of LSD in indigenous case farms	43	691	638	50	4,000
	Cost of treatment of LSD in exotic case farms	59	2,442	2,604	150	12,000
	Cost of vaccination of LSD in indigenous case farms	132	42	9	30	50
	Cost of vaccination of LSD farms with exotic breeds	131	65	39	20	120

The mean cost of cattle mortalities during LSD outbreaks was estimated at 3,103 KSH/31 USD (range 180–6,026 KSH/2–60 USD) per farm keeping indigenous breeds and 43,557 KSH/431 USD (range 3,000–63,900 KSH/30–633 USD) per farm keeping exotic breeds (*P* = 0.04) as shown in Figure 2 and Table 3. The equivalent on a per affected animal basis was 55 KSH/0.5 USD (8–102 KSH/0.1–1 USD) for farms having indigenous cattle and 9,485 KSH/94 USD (range 500–19,133 KSH/5–189 USD) for farms having exotic breeds of cattle (*P* = 0.11) (Figure 3, Table 4).

The indirect costs incurred for treatment of clinically affected cattle in farms keeping indigenous cattle was estimated at a mean of 3,715 KSH/37 USD compared to 5,003 KSH/50 USD for farms keeping exotic breeds (*P* = 0.30). The cost of vaccination against LSD per farm was estimated at 1,117 KSH/11 USD and 178 KSH/2 USD for indigenous (*n* = 5) and exotic (*n* = 8) farms, respectively, that vaccinated cattle before the LSD outbreak (*P* = 0.07) and 888 KSH/9 USD and 852 KSH/8 USD for farms with indigenous and exotic breeds of cattle, respectively, for reactive vaccination (*P* = 0.96) (Figure 2, Table 3). The preventive vaccination in farms with indigenous breeds was higher than those of the farms with exotic breeds owing to the relatively larger herd sizes in the farms with indigenous breeds. The costs of treatment per animal was 691 KSH/7 USD (range 50–4,000 KSH/0.5–40 USD) for indigenous breeds of cattle and 2,442 KSH/24 USD (range 150–12,000 KSH/1–119 USD) for exotic breeds of cattle (Figure 2, Table 3). Vaccination costs per animal were 42 KSH/0.4 USD (range 30–50 KSH/0.3–0.5 USD) for indigenous breeds of cattle and 65 KSH/0.6 USD (range 20–120/0.2–1.1 USD) for exotic breeds of cattle (Figure 3, Table 4).

Based on these estimates, the overall mean loss due to LSD for farms keeping indigenous cattle was estimated at 12,431 KSH/123 USD compared to 76,297 KSH/755 USD for farms keeping exotic breeds of cattle. The analysis indicated that farms keeping indigenous breeds of cattle incurred huge costs through milk and mortality losses respectively, yet they could have been prevented through implementing preventive vaccination. This would have cost approximately a mean of 521 KSH/5 USD per herd with savings amounting to 4,204 KSH/42 USD for farms keeping indigenous breeds of cattle and 43,036 KSH/426 USD for farms keeping exotic breeds of cattle assuming the vaccine is 100% effective. This level of savings in farms could be reallocated to other disease control efforts within these farms.

## Discussion

The results of this study revealed that smallholder farms in Nakuru County, Kenya, are at an increased risk of LSD if they own exotic breeds of cattle and if they source their replacement animals from outside the herd. Economic losses from LSD were also greater in exotic cattle than in indigenous cattle. An assessment of the overall economic impact of LSD on affected farms indicated that those owning exotic breeds of cattle had a higher impact at 76,297 KSH/755 USD compared to those owning just indigenous cattle at 12,431 KSH/123 USD. On both exotic and indigenous breed farms, most of the losses were direct from reduced production.

The results from this study indicate that herds with indigenous cattle were at lower risk of disease compared to those with exotic breeds. Differences in management practices could partly explain this observation and residual confounding cannot be discounted. However, there are numerous reports that indigenous (i.e., *Bos indicus*) cattle appear to be at a lower risk and to have less severe clinical signs compared to exotic breeds. This includes the findings of Khalafalla et al. (44) who observed more severe clinical signs of LSD in exotic cattle in Sudan. In this context, exotic (i.e., *Bos taurus*) breeds are mainly based on Holstein-Friesian and occasional Jersey, Ayrshire, or Guernsey genetics. The relative increased susceptibility of exotic breeds has been reported for several diseases endemic to East Africa including foot-and-mouth disease (45), theileriosis (46, 47), and tuberculosis (48). The reason for this is likely genetic with differences in the innate immune responses between these species (49). In the case of LSD, it is unknown if the incidence of infection varies between species and if subclinical infection is more common in indigenous breeds which would require further studies either in experimental or field settings. There could also be differences in the susceptibility to ectoparasites that spread infection (50). The indigenous breeds of cattle may have the benefit of prolonged periods of natural selection favoring individuals with greater resistance to local diseases although this may not be relevant for LSD which was first recognized in Kenya in 1957 (38) which is not far from the time exotic breeds were introduced into Kenya in 1902 (51, 52).

Obtaining replacement stock from outside the farm was associated with an increased risk of LSD. This is in accordance with studies from Ethiopia (9) and Europe (16). This association is likely due to introduction of virus through infected animals that may have been within the incubation period of the disease and thus not showing clinical signs, subclinically infected or demonstrating clinical signs and purchased at a reduced price. Practices that encourage mixing of cattle such as communal grazing and watering, were not significantly associated with LSD outbreaks which is consistent with observations by Zelalem et al. (8) in the West Wolega zone of Ethiopia but in contrast to the study by Gari et al. (9). These differences may have been due to other differences in herd management and study designs.

Animal movements for reasons of trade and searching for pasture and water during the dry season is also considered to be a risk factor of LSD (17, 18). These aspects were not investigated in the current study which focussed on sedentary herds. Although most of the farmers did not have any understanding of the reasons for LSD occurring on their farm, some farmers believed that the reasons were mixing of affected cattle with unaffected ones during roadside grazing, spread by the wind, spread by biting flies, direct spread from the initial case that occurred in the area, cattle being introduced from other parts of the country and pastoralist cattle in search of pasture. Movements of pastoralist herds in Nakuru County is common and they are frequently blamed for introducing other diseases such as foot-and-mouth disease (53) which can create conflict between communities. Their role should be investigated, and measures designed to reduce any associated impacts from these movements such as enhanced surveillance strategies and ensuring quality vaccines are available to herds at high risk of exposure.

There were some instances when vaccinated farms reported disease and there was no observed difference in the odds of vaccination between case and control herds indicating limited effectiveness against disease. However, this observation should be treated with caution as detailed vaccination history was not available, and the study was neither powered nor designed to measure this effect. Using standardised terminology from vaccine studies in humans, vaccine effectiveness compares the incidence of an outcome between vaccinated and non-vaccinated groups as measured under programme conditions adjusted for exposure risk (54). This differs from vaccine efficacy which is measured under ideal conditions usually in the form of a randomised controlled trial. Case-control or cohort approaches can be used to measure vaccine effectiveness with the former preferred in situations where the outcome incidence is low (55). For LSD, vaccines are either based on LSD virus (homologous) or sheep and goat pox viruses (heterologous). Various efficacy studies mainly based on small-scale challenge studies have been published which generally indicate homologous vaccines confer higher rates of protection compared to heterologous although one study provided some evidence of comparable efficacy using the Gorgan goat pox strain (56, 57). Analysis of the recent LSD outbreak in Europe in 2014–2018 provided evidence that vaccination programmes based on the Neethling strain of LSDV were very effective at controlling the disease (32, 58). A randomised field trial in Israel also demonstrated relative superior efficacy of the homologous vaccine (59). The vaccine available in Kenya was previously believed to be heterologous although molecular studies revealed it to be a homologous virus and there have been numerous reports of poor efficacy and effectiveness (56, 57, 60). Rigorously designed field effectiveness studies should be prioritised and suboptimal performance thoroughly investigated so that limited resources allocated by farmers and governments to LSD control are appropriately used.

The economic impact of LSD in this study was higher for farms with only exotic cattle compared to indigenous cattle. This is consistent with the findings from a recent study from Ethiopia (37) where it was found that the mortality loss, which is a major contribution to financial loss, was higher in exotic crossbreeds compared to the local breeds. For herds with exotic breeds, the higher direct costs are ascribed to higher losses due to reduced milk production and higher mortality. The same study from Ethiopia also revealed these to be the largest components of economic loss due to LSD on affected farms. This is consistent with the previously mentioned studies highlighting the greater severity of disease in exotic breeds. In a study of constraints of cattle production in pastoral areas, where indigenous breeds predominate (61), LSD was found to be a disease with low score for impact on livelihoods (5%) and low incidence (3%). It was ranked eighth among the 13 prevalent diseases in pastoral areas (62).

Milk production due to LSD dropped from a mean of 11.9 and 4.0 l per farm per day to 2.0 and 2.5 l per farm per day, an 83.2 and 37.5% drop for farms keeping exotic and indigenous cattle, respectively. In a study conducted in Ethiopia, milk reduction was by 5 l per cow per day (60). In another study in Kenya, milk production dropped by more than 50% attributed to fever and general sickness in both indigenous and exotic breeds who were found to be equally susceptible to the disease (17). The losses are higher in more severe cases of disease which appears more likely in exotic breeds which has been attributed to their relatively thin skin compared to the thick-skinned indigenous breeds (63). Treatment costs were higher at the individual animal level for exotic breeds which could be due to higher required doses of medicines from a greater liveweight, but also faster recovery in indigenous cattle. There may also be a tendency of herds owning exotic breeds to seek advice and receive visits from animal health service providers compared to those owing indigenous breeds that may tend to deal with diseases themselves and purchase medicines over the counter. Secondary bacterial infection is responsible for most of the illness and loss of production in herds indicating a possible role for antimicrobials (17) although their prudent use is fundamental due to issues with anti-microbial resistance and may not be necessary particularly in indigenous breeds. This suggests a study assessing the efficacy of different treatment protocols for LSD would be worthwhile and to the authors' knowledge has not been previously published.

There are numerous limitations to this study that should be highlighted. A major limitation was the lack of reliable farm-level data which were based on farmer interviews and like other businessmen, farmers may have the tendency to exaggerate their losses. Additionally, as with all case-control studies, there was possible bias from control selection although efforts were made to ensure this was random. Selection bias may also exist from under-reporting of disease. With the study being retrospective, it was not possible to establish causality while recall bias, whereby people interviewed on case and control herds remember events differently, cannot be ruled out or quantified. Ideally, incident cases and confirmation of cases is recommended, but available resources did not allow such data to be collected. Therefore, any reported associations should be treated with caution. For the economic analysis, the focus was just on direct losses from mortality and milk production, and indirect losses from treatment and vaccines. There are numerous other impacts that were not quantified such as the impact on growth rates, fertility, or changes in herd structure (64) so the estimates presented in this study are likely to be lower than the true economic cost. From a study that was carried out to estimate the returns from smallholder dairy farming in Nakuru, it was reported that the net returns per litre of milk produced was 3.6 KSH/0.05 USD (65). Therefore, both the direct and indirect costs incurred by cattle producers due to occurrence of LSD within their herds would reduce this level of net benefits from dairy farming.

In conclusion, the results of this study indicate that farms with exotic breeds of cattle and sourcing of replacement stock from outside are the major risk factors for spread and maintenance of LSD in the Nakuru county. Although vaccines were not associated with a reduced incidence of disease, further studies are required to more rigorously evaluate their effectiveness and also the potential benefits of other control measures such as post-purchase quarantine. The relatively high impact among herds owning exotic breeds indicates that these herds would benefit more from ensuing the availability of quality vaccines and access to appropriate medications. These findings could be used as the basis for developing extension materials to train farmers and animal health service providers as well as supporting the allocation of resources by the veterinary authorities responsible for disease control in Nakuru County.

## Data Availability Statement

The datasets generated for this study are available on request to the corresponding author.

## Ethics Statement

The study was reviewed and approved by Biosafety, Animal Care and Use committee, Nairobi, Kenya.

## Author Contributions

SK, PK, JO, and NL contributed conception and design of the study, data analysis, and interpretation. SK collected the data, organized and did statistical analysis, and prepared the manuscript. PK, JO, and NL supervised the proposal preparation, presentation, and data collection. SK, PK, JO, PB, and NL read, revised, and approved the submitted version of the manuscript.

## Conflict of Interest

The authors declare that the research was conducted in the absence of any commercial or financial relationships that could be construed as a potential conflict of interest.

## Abbreviations

GCRF: Global Challenges Research Fund
BBSRC: Biotechnology and Biological Sciences Research Council
FAO: Food and Agriculture organization of the United Nations
KNBS: Kenya National Bureau of Statistics
LSD: Lumpy Skin Disease
AU-IBAR: African Union Inter-African Bureau for Animal Resources
EFSA: European Food Safety Authority
OIE: World Organization for Animal Health
CFSPH: Centre for Food Security and Public Health
SCVO: Subcounty Veterinary Officer
STATA: Data Analysis and Statistical Software
AI: Artificial Insemination
Ksh.: Kenya Shillings
USD: United States of America Dollars
CI: Confidence Interval
OR: Odds Ratio
LSDV: Lumpy Skin Disease Virus
GALVmed: Global Alliance for Livestock Veterinary Medicines
MS: Microsoft
LRT: Likelihood Ratio Tests
FVM: Faculty of Veterinary Medicine
BAUEC, Biosafety: Animal Care and Use Committee.
