# Supplementary material for: Risk Factors for Outbreaks of Lumpy Skin Disease and the Economic Impact in Cattle Farms of Nakuru County, Kenya
Source: Front Vet Sci. 2020 May 29;7:259. doi: 10.3389/fvets.2020.00259 (PMC7274042; doi:10.3389/fvets.2020.00259)
Supplement: Supplementary file 1 [file Data_Sheet_1.PDF]

Questionnaire on risk factors for LSD and the economic impact of the disease in Nakuru County, Kenya.

Section A: INTRODUCTION

Date: ________Subcounty: _________Sub-Location: _________Village: ________

Section B: FARM INFORMATION

1. What is the relationship of the respondent to the farm?

   1= Owner 2 = Manager 3= Other (Specify) ________________

2. How do you control ticks?

   1= Home spraying 2 = Communal cattle dip 3= Other (Specify) _______________

3. What is the frequency of acaricide dipping/spraying?

   1= Twice a week 2 = Once a week 3= Biweekly 4= Other (Specify) ______________

4. How many cattle in total (plus calves) do you currently own? ________________

5. What breeds and number of cattle do you own in each of the following categories?

Breed	No. Lactating	No. female calves	No. Male calves	No. heifers	No. bulls	No. dry cows

6. Has there been an outbreak of LSD in this farm? (You may refer to the pictures for clinical presentation of LSD) ________________

   1= Yes; 2=No

7. If yes, when did the LSD outbreak occur? ________________

8. What clinical signs of the Lumpy Skin Disease were seen/observed?

____

____

____

How many cattle contracted the disease during the outbreak? ______________

9. What was the total number of cattle that died due to the disease outbreak? _________

10. What was the total number of cattle that recovered from the disease? ____________

11. What was the total number of unaffected cattle? ________________

12. For the cattle that died, what breed(s) and number were affected according to the following categories?

Breed	No. Lactating	No. female calves	No. Male calves	No. heifers	No. bulls	No. dry cows

13. For the cattle that died of LSD and were lactating, how much milk in litres were you getting from them before they died of LSD? ________________

14. For the cattle that recovered from LSD and were lactating, how much milk in litres per day were you getting from them before they were affected by LSD? ___________

15. How much milk in litres per day were you getting from the cows after they were affected by LSD? ____________

16. How much milk in litres per day were you getting from the cows before the LSD outbreak in the farm? ________________

17. Has there been any introduction of new cattle into the herd since Sept last year? ___

1=Yes; 2= No

18. If yes, when was it? (mm/yy) ________________

19. What was the purpose of the introduction? Select as many as applicable

1=Replacement animal; 2=Increasing the herd 3=Bull service

4= Other (Please specify) __________________

20. How many animals were introduced to the farm?

21. Where did the cattle come from? ________________

22. Have you ever carried out vaccination against LSD since 2016? ________________

1=Yes; 2=No

23. If yes, when was it carried out? ________________

24. How many animals were vaccinated? ______________

25. What was the cost of vaccination (Ksh/animal)? ________________

26. Did any of the vaccinated cattle contract LSD? ________________

1= Yes; 2=No

27. If no vaccination was carried out, what was the reason for not vaccinating? ________________

28. Where do you always graze your cattle (Tick as many as possible)?

1. Grazing along the feeder roads

2. Grazing along the main roads

3. Grazing in shared post-harvest fields

4. Grazing in private land

5. Grazing in shared land

6. Zero-grazing

7. Tethering

8. Forest

9. Game reserve/park/conservancy

10. Other (Please specify) ____

29. Where do you always water your cattle (Tick as many as possible)?

1. Shared river

2. Shared dam/pond

3. Private access to river

4. Private dam/pond

5. Own borehole

6. Piped water

7. Harvested rain water

8. Other (Please specify) ____

30. What factor(s) do you think may have contributed or contributes to the disease outbreak in this area?

____

31. How many cattle that contracted LSD were treated (for secondary bacterial infection and wounds etc)? _________________

32. How much did you spend on the treatment of LSD per animal? _________________

33. Which means of breeding do you use? *1. Artificial insemination* *2. Bull*
